# Supplementary material for: New Insight into the Genome-Wide Diversity and Admixture of Six Colombian Sheep Populations
Source: Genes (Basel). 2022 Aug 9;13(8):1415. doi: 10.3390/genes13081415 (PMC9407362; doi:10.3390/genes13081415)

<u>Supplementary Figure S1</u>: Model complexity obtained using coefficient of cross validation of Admixture software (cv) estimates.

A) results for global dataset

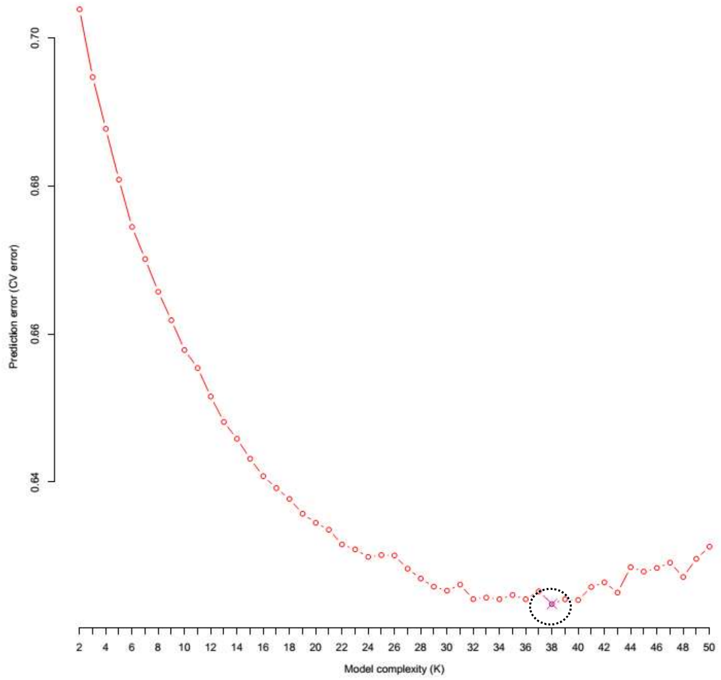

B) Results for Colombian sheep only.

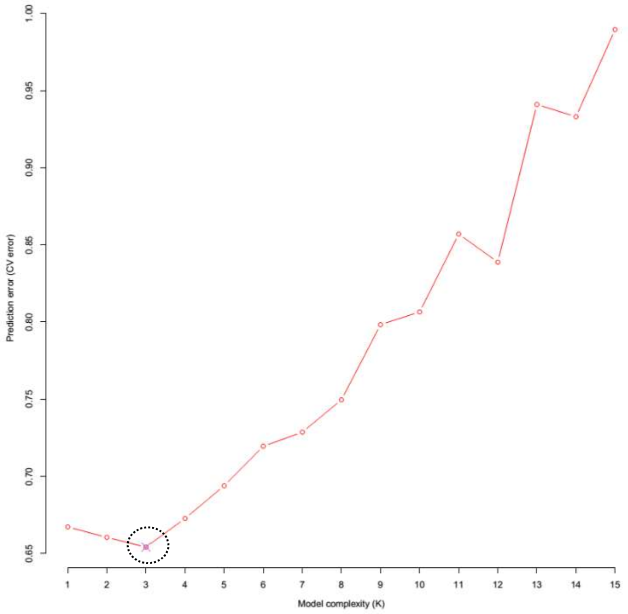

Supplement: Supplementary file 1 [file genes-13-01415-s001.zip › Supplementary_Figure_S1.pdf]
