# Supplementary material for: New Insight into the Genome-Wide Diversity and Admixture of Six Colombian Sheep Populations
Source: Genes (Basel). 2022 Aug 9;13(8):1415. doi: 10.3390/genes13081415 (PMC9407362; doi:10.3390/genes13081415)

Supplementary Figure S2: Netview estimation of most likely community starting point using the "*plotSelection"* option with *Fast-gredy* and *Infomap* algorithm. Arrows indicated the two points chosen for plotting the results.

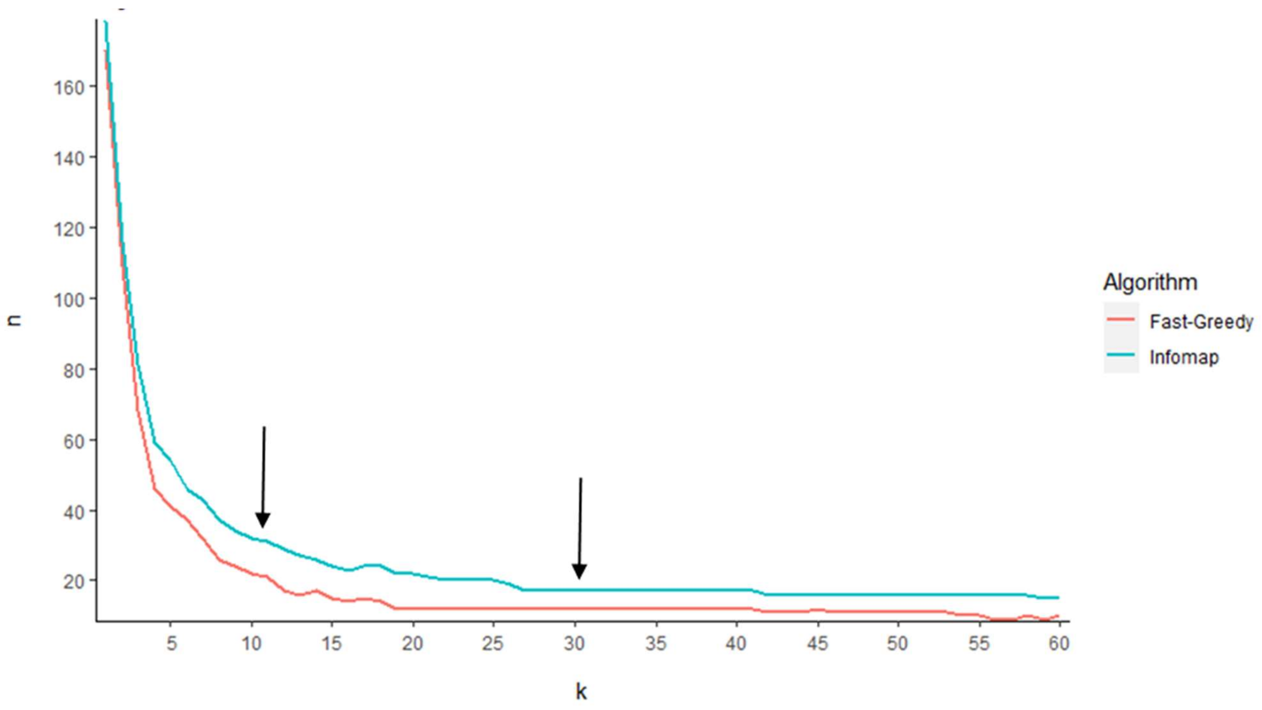

Supplement: Supplementary file 1 [file genes-13-01415-s001.zip › Supplementary_Figure_S2.pdf]
